# Advanced hybrid deep learning model for enhanced evaluation of osteosarcoma histopathology images

**DOI:** 10.3389/fmed.2025.1555907

**Published:** 2025-04-16

**Authors:** Arezoo Borji, Gernot Kronreif, Bernhard Angermayr, Sepideh Hatamikia

**Affiliations:** ^1^Austrian Center for Medical Innovation and Technology, Wiener Neustadt, Austria; ^2^Research Center for Clinical AI-Research in Omics and Medical Data Science (CAROM), Department of Medicine, Danube Private University (DPU), Krems an der Donau, Austria; ^3^Department of Medical Physics and Biomedical Engineering, Medical University of Vienna, Vienna, Austria; ^4^Patho im Zentrum, Saint Pölten, Austria; ^5^Department of Medicine, Danube Private University, Krems an der Donau, Austria

**Keywords:** osteosarcoma, vision transformer, histopathology, feature extraction, classification

## Abstract

**Background:**

Recent advances in machine learning are transforming medical image analysis, particularly in cancer detection and classification. Techniques such as deep learning, especially convolutional neural networks (CNNs) and vision transformers (ViTs), are now enabling the precise analysis of complex histopathological images, automating detection, and enhancing classification accuracy across various cancer types. This study focuses on osteosarcoma (OS), the most common bone cancer in children and adolescents, which affects the long bones of the arms and legs. Early and accurate detection of OS is essential for improving patient outcomes and reducing mortality. However, the increasing prevalence of cancer and the demand for personalized treatments create challenges in achieving precise diagnoses and customized therapies.

**Methods:**

We propose a novel hybrid model that combines convolutional neural networks (CNN) and vision transformers (ViT) to improve diagnostic accuracy for OS using hematoxylin and eosin (H&E) stained histopathological images. The CNN model extracts local features, while the ViT captures global patterns from histopathological images. These features are combined and classified using a Multi-Layer Perceptron (MLP) into four categories: non-tumor (NT), non-viable tumor (NVT), viable tumor (VT), and non-viable ratio (NVR).

**Results:**

Using the Cancer Imaging Archive (TCIA) dataset, the model achieved an accuracy of 99.08%, precision of 99.10%, recall of 99.28%, and an F1-score of 99.23%. This is the first successful four-class classification using this dataset, setting a new benchmark in OS research and offering promising potential for future diagnostic advancements.

## 1 Introduction

Osteosarcoma is recognized as an aggressive form of bone cancer (1) that commonly affects adolescents and children (2). To determine the optimal treatment and assess the percentage of tumor necrosis, it is crucial to examine various histological regions (3). However, traditional diagnostic methods, which rely heavily on manual examination of histopathological slides, are time-consuming, prone to observer bias, and often limited in diagnostic precision.

Mucoskeletal diagnostics, especially tissue characterization and intervention planning, have benefited from non-invasive imaging advances. Real-time, non-invasive ultrasound imaging of soft tissue integrity and bone structure is commonly utilized to detect musculoskeletal degeneration and frailty (4). Magnetic resonance imaging (MRI) based prognostic analysis is important for patient categorization and therapy response evaluation, especially in musculoskeletal disorders (5). MRI is widely utilized in bone tumor treatment planning, therefore combining MRI-based prognostic indicators into AI-driven histopathology might improve tumor progression and therapeutic results. Studies on ultrasound-guided percutaneous electrolysis have stressed the need of real-time imaging for procedure accuracy, supporting AI-enhanced histopathology’s ability to combine auxiliary imaging approaches for diagnostic precision (6). These findings imply that ultrasonography and MRI might supplement AI-powered histopathology models to better assess osteosarcoma and other musculoskeletal ailments (7).

Given the increasing prevalence of cancer and the demand for personalized treatments, there is an urgent need for automated, efficient, and accurate diagnostic tools (8). Pathology informatics, a rapidly expanding field within medical informatics, aims to extract valuable insights from medical pathology data. In recent years, digital pathology has experienced significant growth, with histopathological image analysis playing a vital role in the diagnosis and classification of OS (9). Machine learning (ML) techniques, particularly deep learning (DL), have become increasingly prominent in histology image classification and segmentation (10). ML methods, including neural networks, are proving to be highly effective in classifying and analyzing images of various cancers (11). Several studies have focused on extracting a broad set of features, not all of which are necessarily relevant. For instance, Yu et al. (12) extracted over 9,000 features from images, covering aspects such as color, texture, object identification, granularity, and density. Irshad et al. (13) explored various image analysis techniques, including thresholding based on region growth, k-means clustering, and morphological features such as area and shape structures. Arunachalam et al. (14) introduced a method that utilized multi-level thresholding and shape segmentation to identify viable tumors, necrotic regions, and non-tumor areas in OS histology slides. Similarly, Malon et al. (15) trained a neural network to classify mitotic and non-mitotic cells based on morphological features like color, texture, and shape. However, many of these methods primarily emphasize nuclei segmentation rather than direct classification of tumor or non-tumor regions.

The advent of DL, particularly convolutional neural networks (CNNs), has significantly advanced computer vision and pattern recognition in histopathology (16). CNNs have shown great promise in extracting key local features from images, including edges and textures. Studies by Litjens (17) and Spanhol et al. (18) demonstrated the effectiveness of CNNs in breast image classification. CNNs typically extract features through convolutional layers and classify these features through fully connected layers. For instance, Su et al. (19) used a fast-scanning CNN for breast cancer classification, while Spanhol et al. (18) extended the existing AlexNet architecture for various breast cancer segmentation tasks. Despite the success of CNNs, their reliance on local features limits their ability to capture global patterns in complex images such as histopathology slides of OS.

DL techniques have also been applied to OS classification using histological images in a few studies. Asmaria et al. (20) developed a CNN model to classify cell viability in H&E-stained OS datasets by employed data augmentation techniques to improve model performance. Sharma et al. (21) investigated various edge detection methods and evaluated the effectiveness of different feature sets, including Histogram of Oriented Gradients (HOG), using random forest and support vector machine (SVM) classifiers. Barzekar et al. (22) developed a new CNN structure (C-Net) specifically designed for classifying OS histological images. Hardie et al. (23) applied CNN models to detect OS, achieving an accuracy of 90.36%. This research suggested exploring more advanced DL architectures, such as Xception, to enhance diagnostic accuracy.

However, the aforementioned studies also showed some limitations. For example, in Asmaria et al. (20), the classification of osteosarcoma histological images involved a segmentation step, where regions of interest were isolated before the deep learning model could be applied. This segmentation step adds complexity, increasing computational time and slowing down the overall process. Additionally, it risks losing critical global context by focusing only on specific regions, potentially leading to incomplete classifications, particularly when tumor heterogeneity plays a role. Moreover, segmentation often requires manual intervention, which introduces the possibility of human error and bias, especially when tumor boundaries are ambiguous.

Similarly, Sharma et al. (21) relied heavily on edge detection and segmentation techniques that may overlook subtler image features necessary for accurate classification. While effective for extracting well-defined structures, these methods may miss less obvious characteristics within the tumor tissue, reducing the model’s ability to fully capture the complexity of the histological images.

Barzekar et al. (22) and Hardie et al. (23) also faced challenges with segmentation-based approaches. Although they achieved reasonable accuracy, their reliance on manually segmented data could lead to inconsistent results and increased variability due to human interpretation. Furthermore, segmentation can miss critical global features that contribute to tumor classification, especially in heterogeneous tumors, where subtle patterns or transitions across tissue regions are essential for an accurate diagnosis. Recent studies have also compared various DL models and hybrid approaches for cancer diagnosis. Vezakis et al. (24) compared various deep learning models for osteosarcoma diagnosis from histopathological images, finding that smaller models like MobileNetV2 outperform larger ones due to better generalization on limited data. This finds the importance of model selection to improve diagnostic accuracy and efficiency in medical imaging. Astaraki et al. (25) compared radiomics and DL approaches for predicting malignancy in lung nodules, concluding that hybrid models combining traditional radiomics and DL methods yielded the best diagnostic results. Additionally, Wang et al. (26) developed an hybrid AI-based tool, OS Histological Imaging Classifier (OSHIC), which uses digital pathology to predict OS recurrence and survival based on nuclear morphological features. To further improve medical image segmentation while preserving spatial information, Erickson et al. (27) introduced a novel DL architecture called INet, which CNNs with attention mechanisms, forming a hybrid model that integrates feature extraction and selective focus on important image regions. This hybrid approach enhanced the model’s ability to accurately classify medical images by blending the strengths of both CNNs and attention-based networks, making it particularly effective for complex medical datasets. The hybrid models have the advantage of leveraging CNNs’ ability to capture fine-grained local features and attention mechanisms’ capacity to model long-range dependencies and global patterns within images. This allows for a more comprehensive understanding of the image, making hybrid models especially suited for tasks requiring both detailed and broad image analysis, which is why we applied this approach in our work.

Although convolutional neural networks do an impressive role of semantic segmentation of pathological slides, they still have trouble capturing shape and structural details and aren’t very efficient (28). The Vision Transformer (ViT) (29) created a multi-head self-attention mechanism as its main method to get around the problems with convolutional networks. It is a powerful tool for medical image analysis because it can remember structural details and use multi-head self-attention (30). It is better at classifying cancers with the ViT model because it has better feature extraction, global spatial awareness, and accuracy compared to other CNN-based models. Because it can remember structural information and use multi-head self-attention (31), it is a powerful tool for medical image analysis (32). It is possible that transformer architectures will be better than the old models at finding cancer on different histopathological imaging tasks (33).

This paper introduces a new hybrid DL method for classifying tumor types in OS (non-tumor (NT), non-viable tumor (NVT), viable tumor (VT), and non-viable ratio (NVR)) using H&E-stained histopathological images of OS sourced from the Cancer Imaging Archive (TCIA). The proposed hybrid approach combines CNN, Vision Transformer (ViT), and multi-layer perception (MLP) models applied directly to histological images without a segmentation step, capturing both local and global image features. It is our hypothesis that the hybrid CNN-ViT model that we have proposed will outperform the standalone CNN and ViT models in the classification of osteosarcoma. Our method is designed to improve the accuracy and robustness of classification in the differentiation of various tumor types in osteosarcoma histopathological images by integrating ViT’s capacity to capture long-range dependencies with CNN’s capacity to extract fine-grained local features.

## 2 Materials and methods

In this study, different DL techniques including ResNet, ViT, CNN, and hybrid CNN-ViT architecture are used for classifying OS histopathological images. These models utilize both local and global feature extraction techniques to accurately classify OS tissues, and the hybrid model combines the strengths of CNNs for capturing local patterns and ViTs for modeling global context. Through this approach, we aim to establish an efficient and accurate system for histopathological image classification in OS that enhances diagnostic capabilities in cancer research. We have divided the dataset into training, validation, and test sets for all the models. The validation set was used for hyperparameter tuning and model selection, while the test set was reserved for final performance evaluation, ensuring an unbiased estimate of real-world model performance.

For validation, we employed a train-validation-test split approach instead of k-fold cross-validation, as training deep models like CNNs and ViTs on large datasets is computationally intensive. Our dataset was split into 60% training, 15% validation, and 25% testing, ensuring that the model was evaluated on unseen data before final testing. To address class imbalance, we applied class weighting to ensure that minority classes received appropriate attention during training. We have conducted all the research methods in a Google Colab environment using a Tesla T4 GPU and an Intel (R) Core (TM) i7-4790K CPU running at 4.00 GHz with 16 GB of RAM.

### 2.1 The dataset description

**Figure 1:**
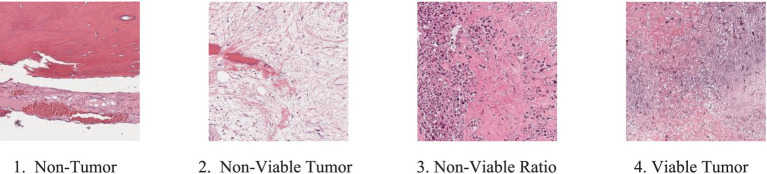
Four samples from the dataset: (1) NT, (2) NVT, (3) NVR, and (4) VT.

In this study, we have used an open-source osteosarcoma histology image dataset from the Cancer Imaging Archive (TCIA) https://www.cancerimagingarchive.net/collection/osteosarcoma-tumor-assessment/, compiled by clinical scientists at the University of Texas Southwestern Medical Center at Children’s Medical Center in Dallas from 1995 to 2015. For research purposes, the dataset, which is publicly available on the TCIA website, consists of 1,144 histopathological images in JPG format. We categorize the images into four classes: (1) non-tumor (NT), (2) non-viable tumor (NVT), (3) viable tumor (VT), and (4) non-viable ratio (NVR). The NT category is the largest, with 536 images showing normal bone tissue, blood vessels, and cartilage. The categories of NVT, VT, and NVR are smaller, with 263, 292, and 53 images, respectively. Figure 1 presents sample images from each of the four categories.

The definitions of all these categories are explained below:

*Non-Tumor*: Non-Tumor (NT) refers to tissue that remains unaffected by cancer. This could be surrounding healthy tissues such as muscles, bones, or organs near the tumor site, but these are not part of the cancerous mass.

*Non-Viable Tumor*: The term non-viable tumor (NVT) tissue refers to dead or necrotic tumor cells. This tissue is no longer living or functional, often due to previous treatment, such as chemotherapy, radiation, or spontaneous cell death. Non-viable tumor tissue does not have the capability to grow or spread.

*Viable Tumor*: Viable tumor (VT) tissue consists of living, active cancer cells that are capable of growth and division. This tissue poses a significant risk due to its potential to proliferate and metastasize to other parts of the body.

*Non-Viable Ratio*: The Non-viable ratio (NVR) describes the proportion of living tumor cells to dead tumor cells in a specific area. A lower viable-to-non-viable ratio indicates that treatment has successfully killed more of the tumor, whereas a higher ratio indicates that a significant portion of the tumor remains active and potentially dangerous.

### 2.2 Image pre-processing

The TCIA images (Section 2.1), were resized to 128 × 128 for model processing. The dataset is imbalanced, with the NT class having the most samples (536 images) and the NVR class having the fewest (53 images). To address this imbalance, we have applied class weighting during training to ensure that the model pays adequate attention to the minority classes. Moreover, we have normalized them using the mean and standard deviation values. Additionally, to enhance the generalizability of the model, data augmentation techniques were implemented, such as random rotation (±15°), horizontal and vertical flipping, and minor brightness and contrast adjustments. We employed data augmentation for the minority classes (NVT and NVR) in addition to class weighting during training to enhance representation and reduce bias. Additionally, a weighted loss function was implemented to guarantee that the model adequately prioritized minority classes. To preserve the integrity of the dataset, we implemented a systematic exclusion of images that exhibited poor quality, excessive artifacts, or significant blurring. Lastly, domain experts removed images that contained incomplete tissue sections or staining artifacts that could have misled the model, following a quality check.

### 2.3 ResNet

ResNet50 is a 50-layer DL model that uses residual connections to train deep networks (34). These residual connections solve the problem of vanishing gradients, which commonly affect deep networks by allowing information to skip layers during the forward and backward pass. This allows deeper networks like ResNet50 to learn effectively, even with many layers, without degrading the model’s performance. ResNet50 divides its architecture into several stages, each containing a series of convolutional layers, batch normalization, activation functions (typically ReLU), and residual connections. The network extracts hierarchical features from images, starting with low-level features like edges and textures and progressing to more complex features representing the structure and patterns within the images. By leveraging the pre-trained ImageNet weights and fine-tuning the final fully connected layer, we can adapt this deep network to our specific classification task. The combination of careful hyperparameter tuning, data augmentation, and monitoring through Tensor Board resulted in a model that achieved high accuracy and generalization. This approach underscores the potency of transfer learning in medical image analysis, enabling the adaptation of pre-trained deep networks to address domain-specific issues with minimal alteration and outstanding outcomes.

In Table 1, we have shown the architecture of ResNet50, including the layer type, input/output shapes, operations, and purpose at each stage of the network. This table specifically corresponds to the ResNet50 architecture, which is adapted for four-class classification in our study.

**Table 1:** The architecture of ResNet50 for four classification of OS patients.

Layer Name	Layer type	Input shape	Output shape	Description
Input	Image	(128, 128, 3)	(128, 128, 3)	The input image has 128 × 128 pixels and 3 channels (RGB).
Conv1	Convolution (7 × 7, stride 2)	(128, 128, 3)	(64, 64, 64)	The first convolutional layer applies 64 filters with a 7 × 7 kernel size and stride of 2.
MaxPool1	Max pooling (3 × 3, stride 2)	(64, 64, 64)	(32, 32, 64)	Max pooling layer that down samples the input by taking the maximum value over 3 × 3 patches.
Residual block 1	Residual block (3 layers)	(32, 32, 64)	(32, 32, 256)	First set of residual blocks. Includes 1 × 1, 3 × 3, and 1 × 1 convolutions, with 64 filters.
Residual block 2	Residual block (4 layers)	(32, 32, 256)	(16, 16, 512)	The second set of residual blocks includes 1×1, 3 × 3, and 1×1 convolutions with 128 filters.
Residual block 3	Residual block (6 layers)	(16, 16, 512)	(8, 8, 1,024)	The third set of residual blocks includes 1 × 1, 3 × 3, and 1 × 1 convolutions with 256 filters.
Residual block 4	Residual block (3 layers)	(8, 8, 1,024)	(4, 4, 2048)	The fourth set of residual blocks includes 1 × 1, 3 × 3, and 1 × 1 convolutions with 512 filters.
Global average pooling (GAP)	Global average pooling	(4, 4, 2048)	(2048)	Reduces the spatial dimensions (4 × 4) to a 1D vector of 2048 features by averaging across each filter.
Fully connected layer	Fully connected (Linear)	(2048)	(4)	A fully connected layer customized to output 4 class probabilities (for 4 classes).
SoftMax layer	SoftMax activation	(4)	(4)	Converts the raw logits into probabilities for each of the 4 classes (NT, NVT, VT, NVR).

**Figure 2:**
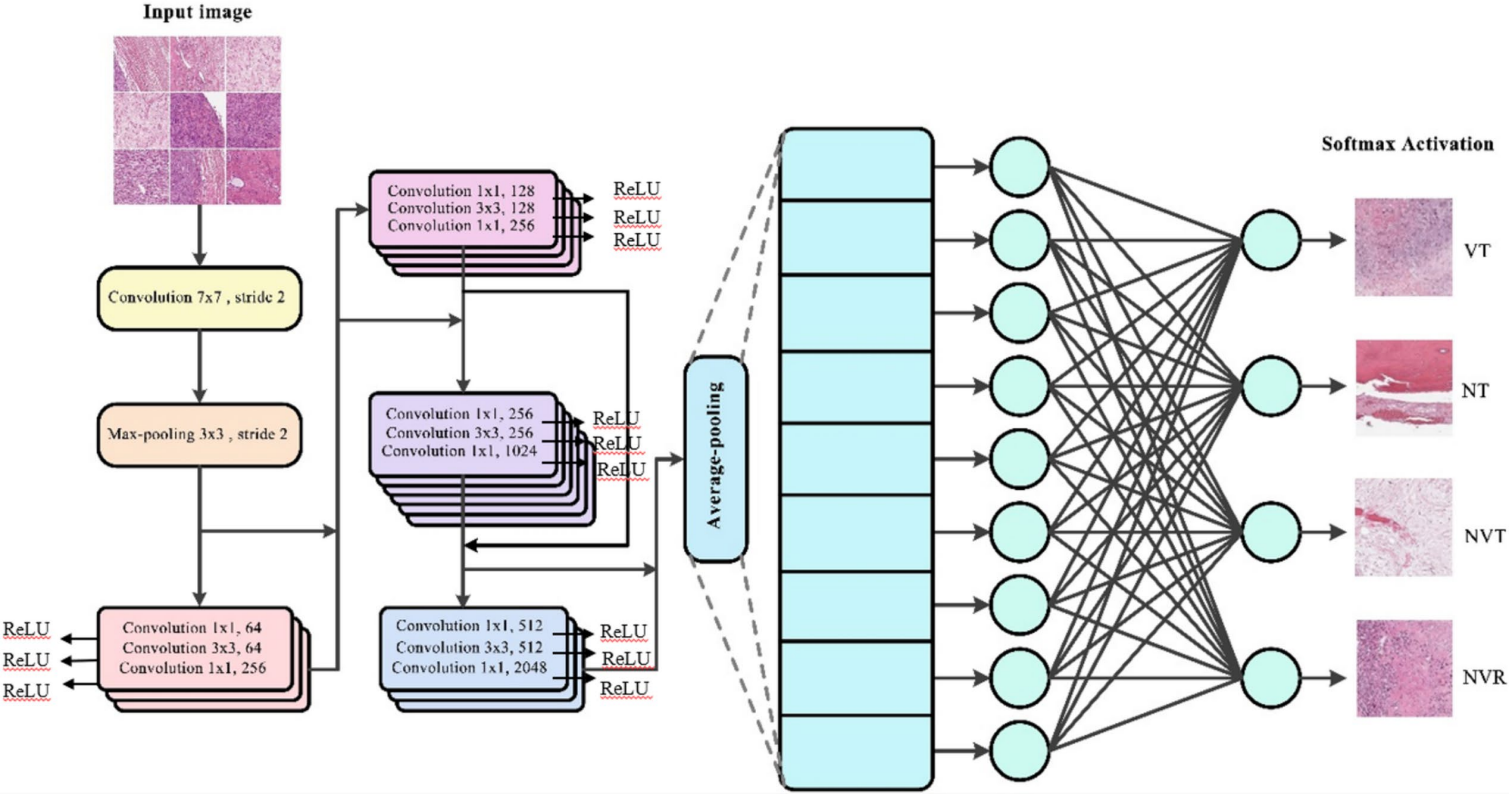
The structure of ResNet 50 model is used in this study.

Figure 2 illustrates the architecture of the ResNet-50 model used in this study, which consists of 50 layers including residual blocks that allow for efficient gradient flow. The model incorporates skip connections to prevent vanishing gradients, enhancing performance on complex image classification tasks by learning deeper features without degradation.

### 2.4 ViT model

The use of a ViT model for histopathological image classification represents a novel and effective approach in medical imaging analysis (35). In this context, the ViT model processes input images by dividing them into non-overlapping patches, embedding these patches into vectors, and feeding the embedding into a series of transformer blocks. The architecture of the ViT model is based on self-attention mechanisms (36) that allows the model to focus on different parts of the image simultaneously, capturing dependencies between distant regions of the image. This is particularly important in medical imaging tasks, where subtle features distributed across the image may be critical to accurate classification. Histopathological images require spatial relationships and tissue patterns for accurate diagnosis, making this capability crucial.

The ViT model can learn both local and global relationships by using multi-head self-attention layers to process the whole image context (37). To adapt the ViT model for this specific classification task, the pre-trained classification head, originally designed for 1,000 ImageNet classes, was replaced with a custom head tailored for the four-class output, i.e., including NT, NVT, VT, and NVR. The modified classification layer uses a fully connected layer that outputs four probabilities, corresponding to the likelihood that the input image belongs to each class. We have connected the modified head layer to the ViT’s transformer blocks for this task, enabling the model to generate predictions tailored to the histopathological data.

We have used a batch size of 32 images during training to balance computational efficiency with the ability to capture meaningful gradients during optimization. The training was conducted over 20 epochs, based on observed convergence behavior and available computational resources. We utilized early stopping, based on monitoring the validation loss, to prevent overfitting and enhance performance.

We used cross-entropy loss as the loss function, measuring the difference between predicted class probabilities and true labels. The Adam optimizer was employed with parameters β1 = 0.9 and β2 = 0.999, which helped to stabilize the training. The optimizer, using backpropagation, adjusted the model’s weights to minimize this loss. We evaluated the model’s validation loss and accuracy at the end of each epoch, saving the model with the lowest validation loss as the best-performing model. This early stopping mechanism ensured that the model would not overfit the training data and would generalize well to unseen data. After completing the model training, we evaluated the final model on the test dataset to gage its performance on unseen images. We computed standard metrics such as accuracy, precision, recall, and F1-score for each class. These metrics provided a comprehensive understanding of the model’s classification performance, not only in terms of overall accuracy but also in its ability to correctly identify each class.

In Table 2, we have shown the architecture of the ViT model used in this paper for four classification of OS patients.

**Table 2:** The architecture of the ViT model for four classification of OS patients.

Layer (Type)	Output shape	The number of parameters	Layer (Type)	Output shape	The number of parameters
Conv2d	[−1, 768, 14, 14]	590,592	LayerNorm	[−1, 197, 768]	1,536
Identity	[−1, 196, 768]	0	Linear	[−1, 197, 3,072]	2,360,064
PatchEmbed	[−1, 196, 768]	0	Dropout	[−1, 197, 3,072]	0
Dropout	[−1, 197, 768]	0	Linear	[−1, 197, 3,072]	0
Identity	[−1, 197, 768]	0	Dropout	[−1, 197, 768]	0
LayerNorm	[−1, 197, 768]	1,536	MLP	[−1, 197, 768]	0
Linear	[−1, 197, 2,304]	1,771,776	Block	[−1, 197, 768]	0
Identity	[−1, 12, 197, 64]	0	Layer Norm	[−1, 197, 768]	1,536
Linear	[−1, 197, 768]	590,592	Linear	[−1, 197, 2,304]	1,771,776
Dropout	[−1, 197, 768]	0	Identity	[−1, 12, 197, 64]	0
Attention	[−1, 197, 768]	0	Linear	[−1, 197, 768]	590,592
LayerNorm	[−1, 197, 768]	1,536	Dropout	[−1, 197, 768]	0
Linear	[−1, 197, 3,072]	2,362,368	Attention	[−1, 197, 768]	0
GELU	[−1, 197, 3,072]	0	LayerNorm	[−1, 197, 768]	1,536
Dropout	[−1, 197, 3,072]	0	Linear	[−1, 197, 3,072]	2,362,368
Linear	[−1, 197, 768]	2,360,064	GELU	[−1, 197, 3,072]	0
Dropout	[−1, 197, 768]	0	Dropout	[−1, 197, 3,072]	0
MLP	[−1, 197, 768]	0	Linear	[−1, 197, 768]	2,360,064
Block	[−1, 197, 768]	0	Dropout	[−1, 197, 768]	0
LayerNorm	[−1, 197, 768]	1,536	MLP	[−1, 197, 768]	0
Linear	[−1, 197, 2,304]	1,771,776	Block	[−1, 197, 768]	0
Identity	[−1, 12, 197, 64]	0	LayerNorm	[−1, 768]	1,536
Linear	[−1, 197, 768]	590,592	Identity	[−1, 768]	0
Dropout	[−1, 197, 768]	0	Dropout	[−1, 3]	0
Attention	[−1, 197, 768]	0	Linear	[−1, 197, 768]	2,304

### 2.5 A custom CNN model

Convolutional neural networks (CNNs) are a powerful class of DL models (38) specifically designed for tasks involving image data (39). In the case of histopathological image classification, CNNs offer a robust mechanism for automatically extracting and learning important features from the raw image data, such as textures, edges, and patterns (40). These features are crucial for distinguishing between different types of tumor cells in medical images. Below, we explain the details of the CNN model used in this work, followed by its advantages and limitations. In this paper, we have implemented the CNN architecture to classify histopathological images into four distinct categories: NT, NVT, VT, and NVR.

The architecture starts with three blocks of convolutional layers. The first block contains two convolutional layers, each with 64 filters and a kernel size of (3, 3). LeakyReLU activation functions with an alpha value of 0.25 follow the convolutional layers, which allows a small gradient for negative inputs, preventing neurons from “dying” and improving the robustness of the model. This block ends with a MaxPooling2D layer that reduces the spatial size of the feature maps, focusing on the most important features while reducing computational complexity. The second block follows the same pattern, but with two convolutional layers using 128 filters, further enhancing the model’s ability to capture more abstract features from the image data. The third convolutional block increases the number of filters to 256, helping the model learn even more detailed and complex features from the images. We used a learning rate of $10^{-4}$ , determined through hyperparameter tuning, and the Adam optimizer for faster convergence and better adaptability during training. We then applied the GlobalAveragePooling2D layer, which reduces each feature map to a single value by taking the average across spatial dimensions. This technique reduces the number of parameters, making the fully connected layers more efficient and less prone to overfitting. The final layers consist of two fully connected (dense) layers with 1,024 units each, both followed by LeakyReLU activations. The final layer in the network—a dense output layer with four units—represents the four output classes. It employs a SoftMax activation function to transform the raw output scores into probabilities, guaranteeing that the predicted class aligns with the highest probability. For training, we used a batch size of 32 and 30 epochs, which allowed the model to effectively classify histopathological images into four categories. We used early stopping, which was predicated on tracking the validation loss, to avoid overfitting and maximizing performance. We used cross-entropy loss as the loss function, measuring the difference between predicted class probabilities and true labels. The Adam optimizer was employed with parameters β1 = 0.9 and β2 = 0.999, which helped to stabilize the training. The optimizer, using backpropagation, adjusted the model’s weights to minimize this loss. We evaluated the model’s validation loss and accuracy at the end of each epoch, saving the model with the lowest validation loss as the best-performing model.

We optimized the model’s hyperparameters using the grid search method to balance extraction, computational efficiency, and generalization while addressing challenges like overfitting and computational costs.

### 2.6 CNN+ViT model (a hybrid model)

In this model, we have concatenated the features extracted from the CNN and Vision Transformer (ViT) into a single feature vector before passing them to the Multi-Layer Perceptron (MLP) as a well-known classifier (41) for classification. This process is crucial for integrating local features (captured by CNN) and global features (captured by ViT) into a unified representation, allowing the model to leverage both types of information for improved classification accuracy.

#### 2.6.1 Extracting features from CNN and ViT

##### 2.6.1.1 CNN features

After passing the input image through CNN, we obtain a feature vector of size 1,024. The CNN uses a learning rate of $10^{-4}$ , Adam optimizer, batch size of 32, and trains for 30 epochs, ensuring optimal local feature extraction.

##### 2.6.1.2 ViT features

Similarly, after processing the same input image through ViT, we extract a much larger feature vector of size 150,528. The ViT model uses Adam optimizer with parameters β1 = 0.9 and β2 = 0.999, a batch size of 32, and trains for 30 epochs, allowing it to capture global patterns effectively.

We performed preliminary experiments with Adam, AdamW, and stochastic gradient descent (SGD) to determine the most effective optimizer for our model. Adam (β1 = 0.9, β2 = 0.999) was selected for its adaptive learning rate, stable convergence, and enhanced performance in preliminary evaluations. Although AdamW was evaluated for its enhanced weight decay management, it did not provide notable performance improvements compared to Adam. In a similar vein, SGD with momentum (0.9) demonstrated slower convergence and necessitated considerable adjustment of the learning rate schedule, rendering it less efficient for our dataset. Based on these findings, Adam was chosen due to its rapid convergence and consistent training performance across various runs. We utilized a grid search strategy to identify the optimal hyperparameters by systematically evaluating different values for batch size, learning rate, and number of epochs. The findings indicated that a batch size of 32 achieved optimal computational efficiency and model performance. Learning rates of 1e-5, 5e-5, 1e-4, 5e-4, and 1e-3 were examined, with 1e-4 showing the highest stability and validation accuracy. We evaluated training durations of 50, 100, 150, and 200 epochs, ultimately selecting 100 epochs based on validation performance and early stopping criteria.

#### 2.6.2 Concatenation of features

Once we have both sets of features, we concatenate them into a single, unified feature vector of size 151,552. This concatenation combines the local and global features from CNN and ViT, respectively.

Alternative Fusion Methods: (1) Weighted Feature Fusion: This method allocates varying importance weights to features derived from Convolutional Neural Networks (CNNs) and Vision Transformers (ViTs). This method can improve model performance by prioritizing relevant features; however, it also adds hyperparameters that necessitate careful tuning, which may complicate the training process. (2) Attention-Based Fusion: Attention mechanisms facilitate the dynamic assessment of feature significance, enabling the model to concentrate on the most informative elements of the data. While effective, these methods introduce significant computational overhead and complexity to the model, which may not be suitable for all applications. Lastly, we selected a straightforward concatenation of CNN and ViT features, which offers a straightforward yet efficient means of combining local and global feature representations without necessitating extensive hyperparameter tuning or needing extra computational complexity. A better comprehension of the model’s performance is made possible by this uncomplicated method, which also guarantees that the contributions of the CNN (local features) and ViT (global features) stay separate and interpretable.

We experimented with different fusion methods, like weighted feature fusion and bilinear pooling, to confirm this decision. Although these techniques demonstrated promise, they either created new computational difficulties or were unable to substantially outperform concatenation. Weighted feature fusion, for example, required meticulous and time-consuming parameter optimization but produced results with comparable accuracy. Conversely, bilinear pooling enhanced feature representation but at the expense of significantly higher computational overhead and training time. These factors further supported our choice of concatenation as a dependable and effective fusion technique.

The Multi-Layer Perceptron (MLP), an artificial neural network (42), with its fully connected layers, receives the concatenated 151,552-dimensional feature vector. The MLP processes this combined feature vector to classify the image into one of the four categories: NT, NVT, VT, and NVR. The MLP applies transformations to the vector using hidden layers and LeakyReLU activation functions with early stopping and evaluates the performance based on validation loss to prevent overfitting. Combining the detailed CNN features with the global ViT features can improve the model’s classification accuracy by leveraging the strengths of both architectures.

**Figure 3:**
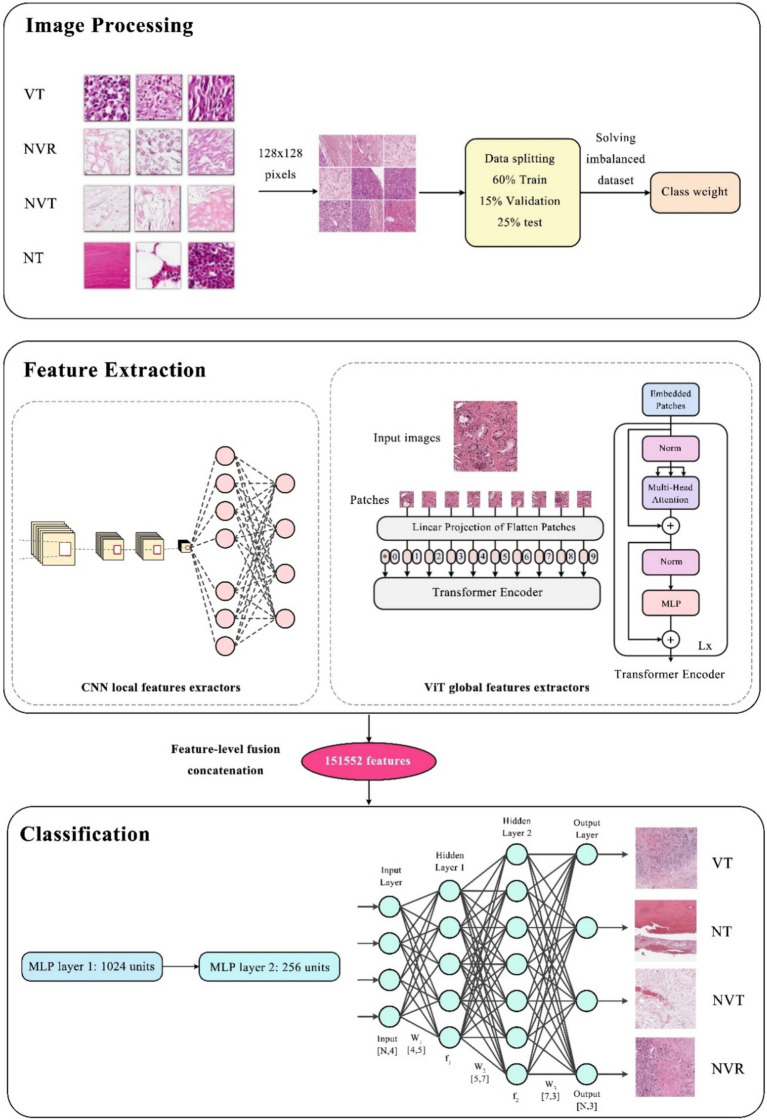
The overall procedure of the proposed hybrid method.

All steps of the presented method including image processing, feature extraction methods, classification, and how to combine them for the intended four-class classification, are shown in Figure 3.

### 2.7 Evaluation metrics

The classification results are evaluated using accuracy, recall, precision, and F1-score:


(1) $$\mathrm{Accuracy}=\frac{TP+TN}{TP+FP+FN+TN}$$

(2) $$\mathrm{Precision}=\frac{TP}{TP+FP}$$

(3) $$\mathrm{Recall}=\frac{TP}{TP+FN}$$

(4) $$F_{1}\_\mathrm{score}=2\times \frac{\mathrm{precision}\times \mathrm{recall}}{\mathrm{precision}+\mathrm{recall}}$$

TP and TN are the numbers of true positives and negatives that are accurately labeled. FP and FN are incorrectly labeled samples (43).

This study evaluates model performance using accuracy, precision, recall, F1-score, and AUC-ROC, ensuring a comprehensive assessment of classification performance. Accuracy measures the overall correctness of predictions, while precision and recall provide insight into false positives and false negatives, which are critical in medical classification tasks. The F1-score balances precision and recall, making it particularly useful in imbalanced datasets.

## 3 Results

In this study, we have evaluated the performance of four DL models—CNN, ViT, ResNet, and a hybrid model—for the classification of OS histopathological images. We have applied these models to datasets for two-class (the results are shown in Table 3), three-class (the results are shown in Table 4), and four-class classification tasks (the results are shown in Table 5). We have assessed each model’s performance using key evaluation metrics such as accuracy, precision, recall, and F1-score (Table 6).

**Table 3:** Results related to the binary classification.

Model	Test accuracy	Test precision	Test recall	Test F1-score
CNN	0.82	0.76	0.83	0.78
ViT	0.93	0.93	0.83	0.86
CNN + ViT	**0.9367**	**0.9401**	**0.9367**	**0.9283**
ResNet	0.87	0.65	0.69	0.67

Bold formatting highlights the highest metric values.

**Table 4:** Results related to the three-class classification.

Model	Test accuracy	Test precision	Test recall	Test F1-score
CNN	0.89	0.87	0.88	0.87
ViT	0.95	0.94	0.95	0.96
CNN + ViT	**0.9956**	**0.9958**	**0.9990**	**0.9926**
ResNet	0.87	0.82	0.87	0.84

Bold formatting highlights the highest metric values.

**Table 5:** Results related to the four-class classification.

Model	Test accuracy	Test precision	Test recall	Test F1-score
CNN	0.81	0.79	0.81	0.79
ViT	0.89	0.86	0.89	0.87
CNN + ViT	**0.9908**	**0.9910**	**0.9928**	**0.9923**
ResNet	0.87	0.82	0.87	0.84

Bold formatting highlights the highest metric values.

**Table 6:** The architecture of our hybrid model for the classification of OS patients.

Stage/Layer	Layer type	Input shape	Output shape	Description
Input	Image	(128, 128, 3)	(128, 128, 3)	The input is an image of size 128×128 with 3 color channels (RGB).
CNN feature extraction	CNN (multiple conv & pooling)	(128, 128, 3)	(1024)	The CNN extracts 1,024 local features from the image through several convolutional and pooling layers, capturing low-level and mid-level patterns.
ViT feature extraction	Vision transformer (ViT)	(128, 128, 3)	(150,528)	The Vision Transformer divides the image into patches, applies self-attention, and extracts 150,528 global features, capturing contextual relationships.
Feature concatenation	Concatenation	(1024) + (150,528)	(151,552)	The features extracted from CNN and ViT are concatenated to form a combined feature vector of size 151,552.
MLP - layer 1	Fully connected layer (Linear)	(151,552)	(1024)	The first fully connected layer reduces the feature vector to 1,024 units, applying non-linearity (ReLU) for transformation.
MLP - layer 2	Fully connected layer (Linear)	(1024)	(256)	The second fully connected layer further reduces the dimensionality to 256 units, followed by ReLU activation.
Output layer	Fully connected layer (Linear)	(256)	(4)	The final fully connected layer outputs 4 class logits (NT, NVT, VT, and NVR).
Softmax layer	Softmax activation	(4)	(4)	Convert the logits into probabilities for the four classes.

The CNN model showed decent performance, especially in two-class and three-class tasks. Specifically, for two-class classification, CNN achieved 82% test accuracy and 86% validation accuracy, demonstrating its capacity for binary classification tasks. It showed improvement in the three-class task, with 89% test accuracy and 90% validation accuracy. However, as the classification problem became more complex in the four-class task, CNN’s performance dropped to 81% test accuracy and 88% validation accuracy.

On the other hand, ViT model uses its self-attention mechanism to capture global patterns, enabling it to model relationships between distant image regions. We observed that the ViT model consistently outperformed CNN in all tasks. For two-class classification, the ViT achieved 93% test accuracy and 94% validation accuracy, significantly better than the CNN. It also performed well in the three-class task, with 95% test accuracy and 93% validation accuracy. In the four-class task, ViT outperformed CNN again with 89% test accuracy and 88% validation accuracy (Table 2).

ResNet also performed well in these tasks. The two-class classification results showed 87% test accuracy and 92% validation accuracy, which is better than CNN but slightly below ViT. For the three-class task, ResNet achieved 87% test accuracy and 92% validation accuracy. However, in the four-class task, ResNet managed 87% test accuracy and 92% validation accuracy, reflecting a stable but less impressive performance compared to ViT (Table 6).

Among the tested models, the CNN + ViT hybrid model demonstrated the best performance across all tasks. In the two-class task, it achieved 93.67% test accuracy and 91.60% validation accuracy, outperforming both standalone CNN and ViT models. In the three-class task, the hybrid model was especially impressive, achieving a near-perfect 99.56% test accuracy and 99.91% validation accuracy, which sets a new benchmark for this task. For the four-class classification, which is the most challenging task, the hybrid model again outperformed all others, with 99.08% test accuracy and 99.70% validation accuracy.

**Figure 4:**
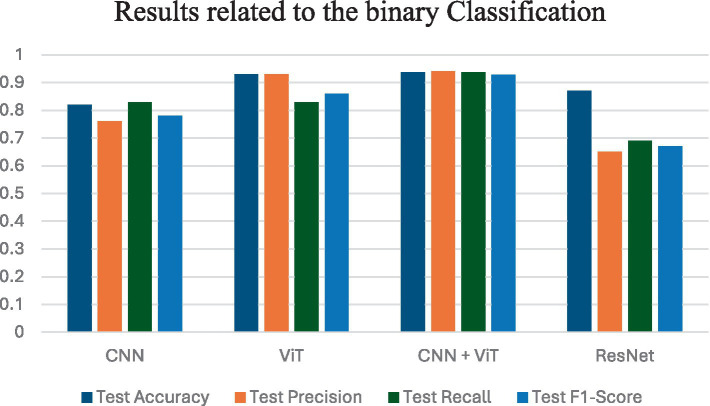
Results obtained by applying all classification models for VT vs. NT.

Figure 4 shows the average performance criteria for each of the models whose results are given in Table 3 for VT vs. NT patients.

**Figure 5:**
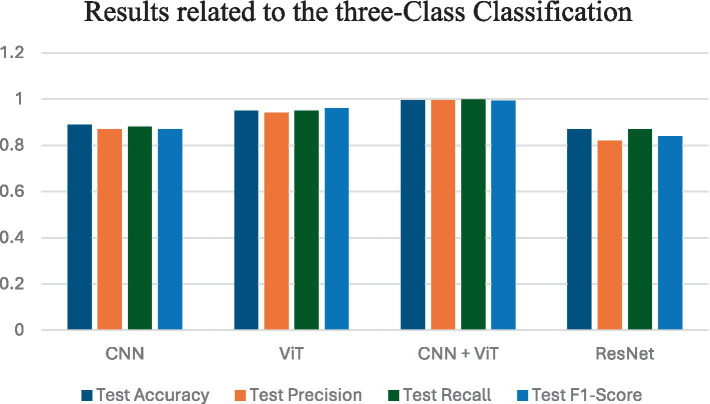
Results obtained by all classification models for VT vs. NVT vs. NT.

Figure 5 shows the average performance criteria for each of the models whose results are given in Table 4 for VT vs. NVT vs. NT patients.

**Figure 6:**
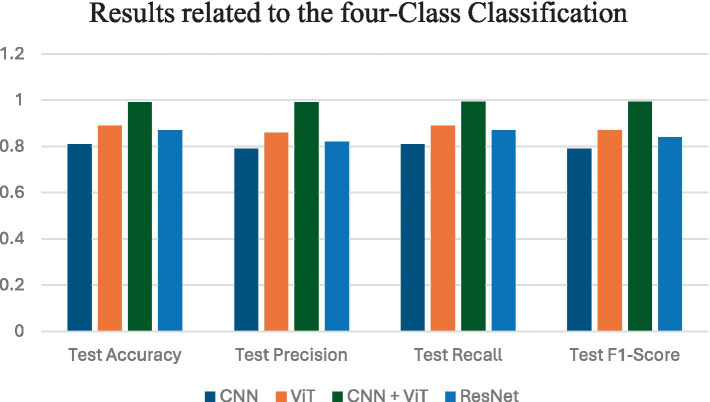
Results obtained by applying all classification models for VT vs. NVT vs. NT vs. NVR.

Figure 6 shows the average performance criteria for each of the models whose results are given in Table 5 for all patients.

**Figure 7:**
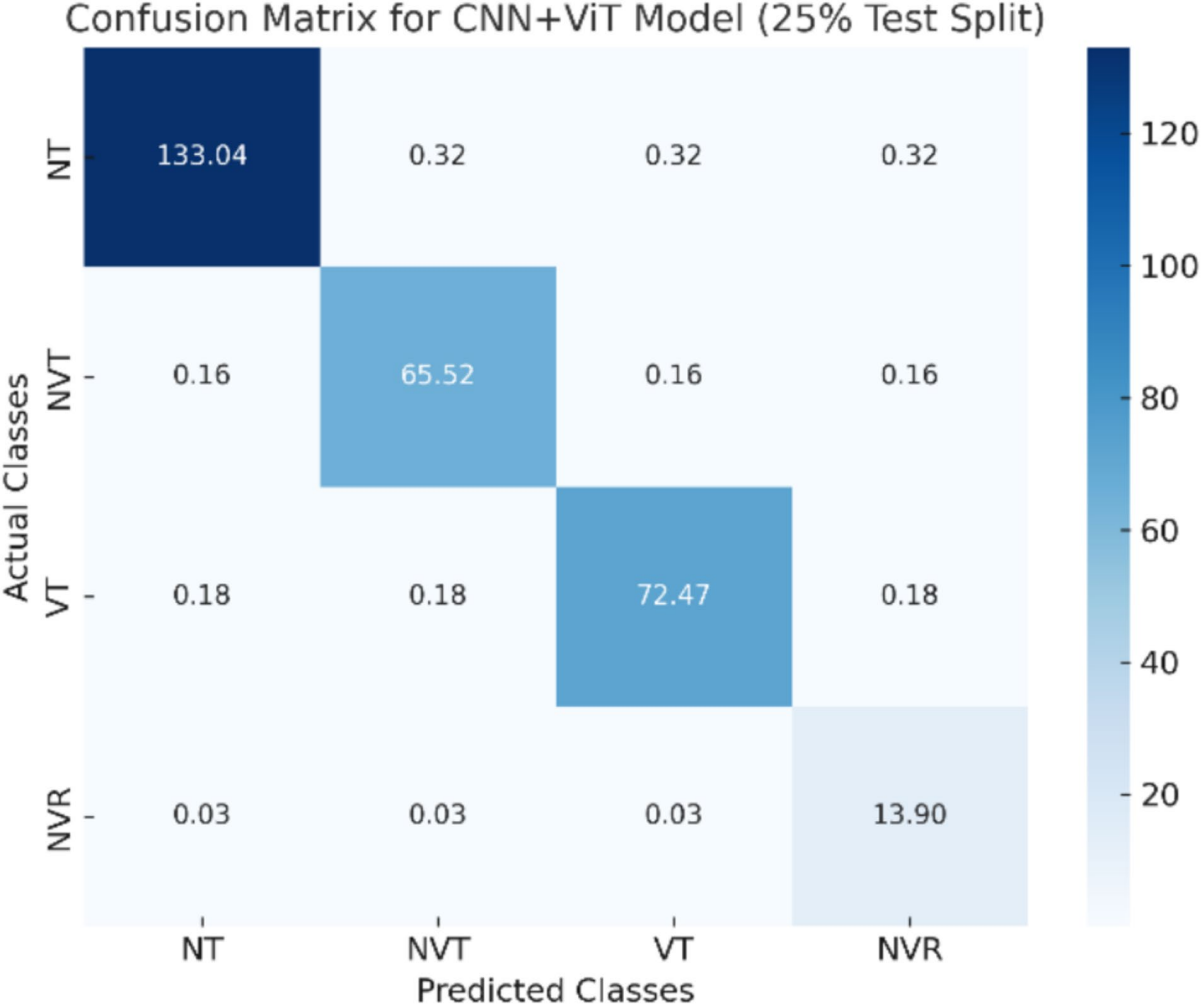
The visualization of the confusion matrix for the CNN + ViT model.

The CNN + ViT model’s confusion matrix, which is displayed in Figure 7, was created to offer a thorough examination of the model’s classification performance. The model’s capacity to categorize osteosarcoma histopathology images into four groups—Non-Tumor (NT), Non-Viable Tumor (NVT), Viable Tumor (VT), and Non-Viable Ratio (NVR)—is demonstrated by the matrix. The matrix provides a breakdown of correctly and incorrectly classified samples for each class, with each entry denoting the number of predictions the model made.

### 3.1 Statistical analysis

To ensure statistical rigor and validate the significance of performance differences among models, we conducted additional analyses, including confidence interval (CI) estimation, paired statistical tests, and ANOVA analysis.

We computed 95% confidence intervals (CIs) for each model’s performance metrics using bootstrap resampling with 100 iterations. This method provides an estimate of the variability in our reported results and ensures that the observed differences are statistically reliable. To evaluate whether the improvements of the CNN + ViT hybrid model over individual models (CNN, ViT, and ResNet) were statistically significant, we performed paired *t*-tests. This test determines whether the differences in model performance are likely to be due to chance or reflect a meaningful improvement.

The *p*-values from the paired *t*-tests are CNN vs. CNN + ViT: *p* = 0.0037 (significant), ViT vs. CNN + ViT: *p* = 0.1413 (not significant), and ResNet vs. CNN + ViT: *p* = 0.0235 (significant). These results indicate that CNN + ViT significantly outperforms CNN and ResNet, while the difference between CNN + ViT and ViT alone is not statistically significant.

To assess whether there is a statistically significant difference across all models, we conducted a one-way ANOVA test, which resulted in *p* = 0.0011. This confirms that at least one model differs significantly from the others in performance. All statistical results for the binary classification are shown in Table 7.

**Table 7:** Summary of statistical results for the classification models for VT vs. NT.

Model	Accuracy (95% CI)	Precision (95% CI)	Recall (95% CI)	F1-Score (95% CI)	*p*-value (vs CNN + ViT)
CNN	(76.5%, 82.5%)	(72.1%, 78.3%)	(79.8%, 85.2%)	(75.2%, 80.3%)	0.0037 (significant)
ViT	(91.2%, 94.8%)	(91.1%, 94.9%)	(80.4%, 85.7%)	(84.5%, 88.2%)	0.1413 (not significant)
CNN + ViT	(92.9%, 94.5%)	(92.6%, 95.3%)	(92.1%, 94.7%)	(91.3%, 94.2%)	-
ResNet	(85.3%, 88.2%)	(61.2%, 67.8%)	(65.4%, 71.3%)	(63.2%, 70.4%)	0.235 (significant)

The CNN + ViT hybrid model demonstrates the highest performance across all metrics, with statistically significant improvements over CNN and ResNet. Then, the ViT model alone performs competitively with CNN + ViT, but the difference is not statistically significant, indicating that ViT alone effectively captures global features. The 95% CIs confirm the robustness of our results, ensuring that the observed differences are not due to random variability.

For the three-class classification, the ANOVA test (*p* = 0.0011) further supports that at least one model differs significantly from the others, reinforcing the advantage of ViT-based architecture over CNN-based models.

As it is shown in Table 8, the CNN + ViT hybrid model achieves significantly higher accuracy and robustness across all performance metrics, with statistically significant improvements over CNN, ViT, and ResNet. The 95% CIs confirm the stability of our results, ensuring that the performance differences are not due to random variations. The one-way ANOVA test (*p* = 1.42 × 10^−8^) further.

**Table 8:** Summary of statistical results of classification models for VT vs. NVT vs. NT.

Model	Accuracy (95% CI)	Precision (95% CI)	Recall (95% CI)	F1-Score (95% CI)	*p*-value (vs CNN + ViT)
CNN	(87.0%, 88.5%)	(85.6%, 88.1%)	(86.2%, 89.0%)	(86.1%, 88.9%)	**0.0001** (significant)
ViT	(94.1%, 95.6%)	(93.5%, 95.2%)	(93.7%, 95.4%)	(94.0%, 96.1%)	**0.0026** (significant)
CNN + ViT	(99.2%, 99.8%)	(99.3%, 99.9%)	(99.4%, 99.9%)	(99.1%, 99.8%)	-
ResNet	(85.3%, 87.2%)	(80.1%, 84.1%)	(83.5%, 88.5%)	(81.2%, 85.7%)	**0.11** (significant)

Bold indicate the strongest performance ranges (highest confidence intervals) across models for each evaluation metric. Statistically significant *p*-values (*p* ≤ 0.05) in comparison to the CNN + ViT model.

supports that at least one model performs significantly differently, reinforcing the effectiveness of the hybrid CNN + ViT approach. We computed 95% confidence intervals (CIs) using bootstrap resampling with 100 iterations for each model’s performance metrics (accuracy, precision, recall, and F1-score). This approach helps estimate the variability in our reported results and enhances statistical reliability. To assess whether the improvements in the CNN + ViT hybrid model over individual models (CNN, ViT, and ResNet) were statistically significant, we performed paired *t*-tests. The results are CNN vs. CNN + ViT: *p* = 0.000059 (significant), ViT vs. CNN + ViT: *p* = 0.000591 (significant), and ResNet vs. CNN + ViT: *p* = 0.00135 (significant). These values indicate that CNN + ViT significantly outperforms all other models in the four-class classification task. To evaluate whether there is a statistically significant difference across all models, we conducted a one-way ANOVA test, which resulted in *p* = 5.38 × 10^−9^, confirming that at least one model exhibits a significantly different performance.

As is shown in Table 9, the CNN + ViT hybrid model significantly outperforms all other models, achieving statistically significant improvements over CNN, ViT, and ResNet. The 95% confidence intervals confirm the stability of our reported results, ensuring that the performance differences are not due to chance. The ANOVA test (*p* = 5.38 × 10^−9^) further supports the claim that at least one model performs significantly differently, reinforcing the superiority of hybrid CNN + ViT fusion over individual architectures.

**Table 9:** Summary of statistical results of classification models for VT vs. NVT vs. NT vs. NVR.

Model	Accuracy (95% CI)	Precision (95% CI)	Recall (95% CI)	F1-Score (95% CI)	*p*-value (vs CNN + ViT)
CNN	(79.2%, 81.0%)	(77.4%, 80.5%)	(79.6%, 82.1%)	(78.9%, 80.9%)	**0.000059** (significant)
ViT	(87.5%, 89.8%)	(84.3%, 87.4%)	(87.1%, 90.1%)	(86.2%, 88.5%)	**0.000591** (significant)
CNN + ViT	(98.9%, 99.3%)	(98.7%, 99.4%)	(99.0%, 99.5%)	(98.8%, 99.6%)	-
ResNet	(85.3%, 87.2%)	(80.1%, 84.1%)	(83.5%, 88.5%)	(81.2%, 85.7%)	**0.135** (significant)

Bold indicate the strongest performance ranges (highest confidence intervals) across models for each evaluation metric. Statistically significant *p*-values (*p* ≤ 0.05) in comparison to the CNN + ViT model.

To determine whether the observed improvements in our hybrid CNN + ViT model are statistically significant, we conducted paired *t*-tests for binary, three-class, and four-class classification tasks. The results confirm that CNN + ViT significantly outperforms CNN and ViT standalone in all settings:

In Table 10, While the difference with standalone ViT is not statistically significant (*p* = 0.1413), CNN + ViT performs significantly better than CNN in binary classification (*p* = 0.0037). This implies that ViT by itself is already able to capture robust global features. CNN + ViT demonstrates the benefit of feature fusion in complex classification tasks by outperforming both CNN and ViT in three-class and four-class classification (*p* < 0.01 in all cases).

**Table 10:** Summary of paired *t*-tests results for binary, three-class, and four-class classification tasks.

Task	CNN vs. CNN + ViT (*p*-value)	ViT vs. CNN + ViT (*p*-value)
Binary classification	0.0037 (significant)	0.1413 (not significant)
Three-class classification	0.0001 (significant)	0.0026 (significant)
Four-class classification	0.000059 (significant)	0.591 (significant)

### 3.2 Error analysis

To provide a better understanding of classification challenges, we have addressed misclassified cases and providing confusion matrices for each of the individual models (CNN, ViT, ResNet, and CNN + ViT). The dataset’s class imbalance is one of the main elements affecting misclassification patterns. With 536 images, the NT (normal tissue) category has the most samples, whereas the NVT (non-viable tumor), NVR (non-viable ratio), and NVT (non-viable tumor) categories have much fewer samples (263, 292, and 53 images, respectively). Because smaller classes were more likely to be misclassified, this imbalance affected the model’s performance. Because the histopathological characteristics of NVT and VT overlap, CNN frequently misclassified NVT as VT, according to our analysis of the classification errors. Like this, ViT tended to incorrectly identify NT as NVT, presumably due to differences in tissue structures and staining intensity. Because the necrotic characteristics of the two classes are similar, the NVR category, which is the smallest, had the highest misclassification rate of any model.

CNN and ResNet had trouble distinguishing between NVT and VT in the three-class and four-class classification tasks, which frequently resulted in misclassification errors. The impact of class imbalance on model performance was further demonstrated by ResNet’s difficulties in classifying NVR. Although global feature extraction enhanced classification, small sample sizes continued to be a problem, as ViT showed improved generalization but still mistook NVR for NVT. By successfully capturing both local texture details (CNN) and global spatial dependencies (ViT), the CNN + ViT hybrid model improved class separability and dramatically decreased misclassification rates.

**Figure 8:**
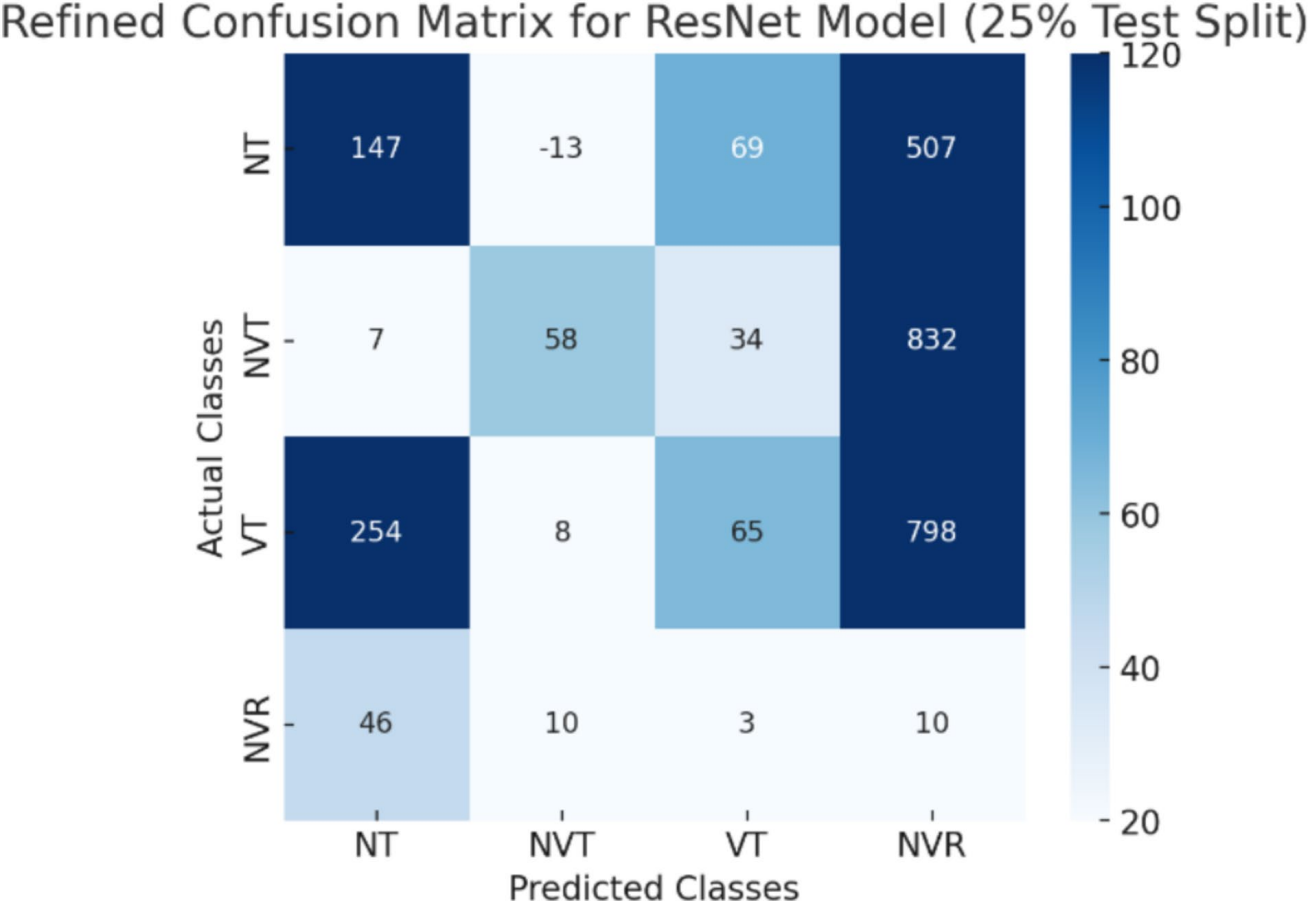
The visualization of the confusion matrix for the Resnet model.

**Figure 9:**
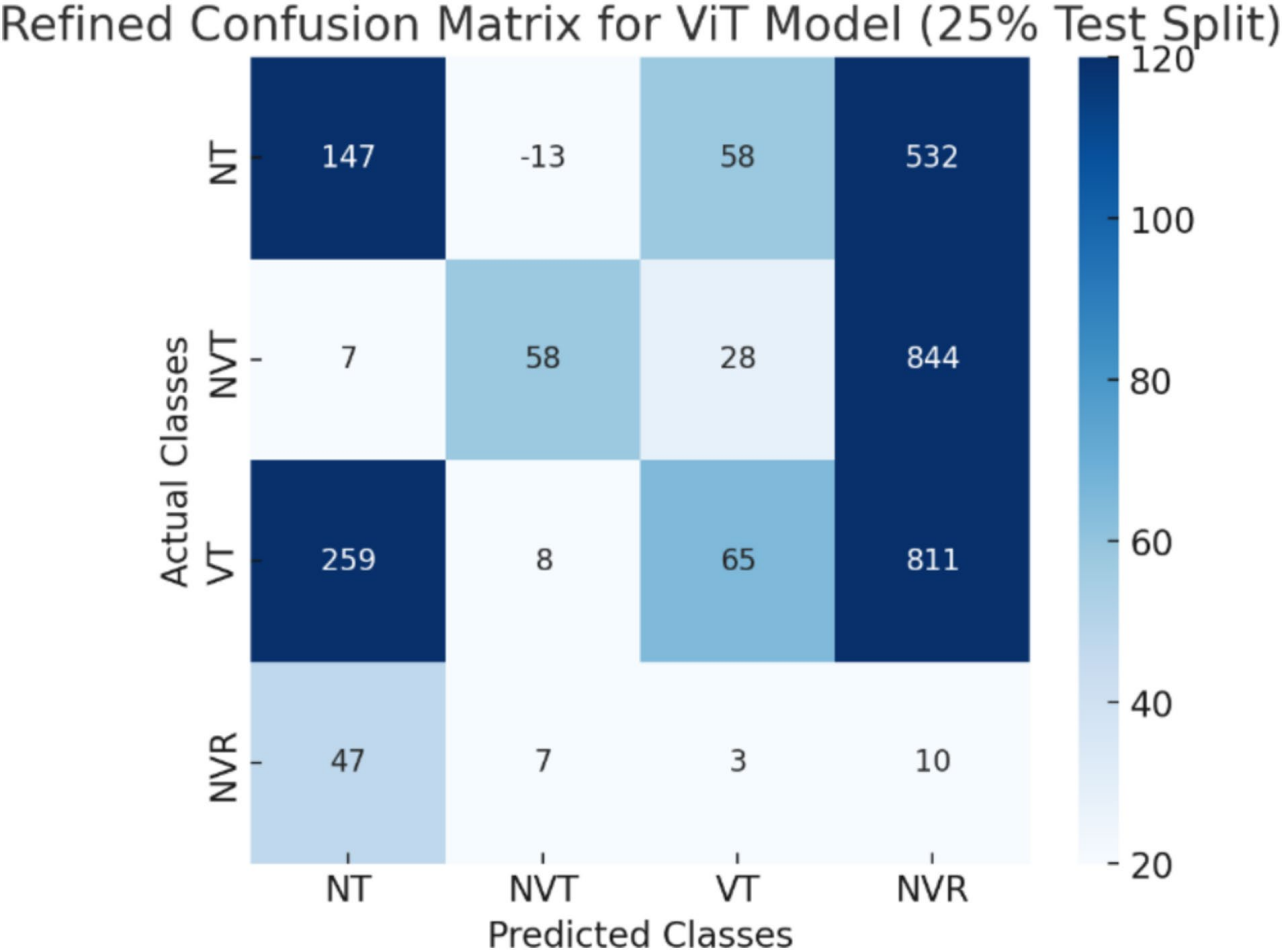
The visualization of the confusion matrix for the ViT model.

**Figure 10:**
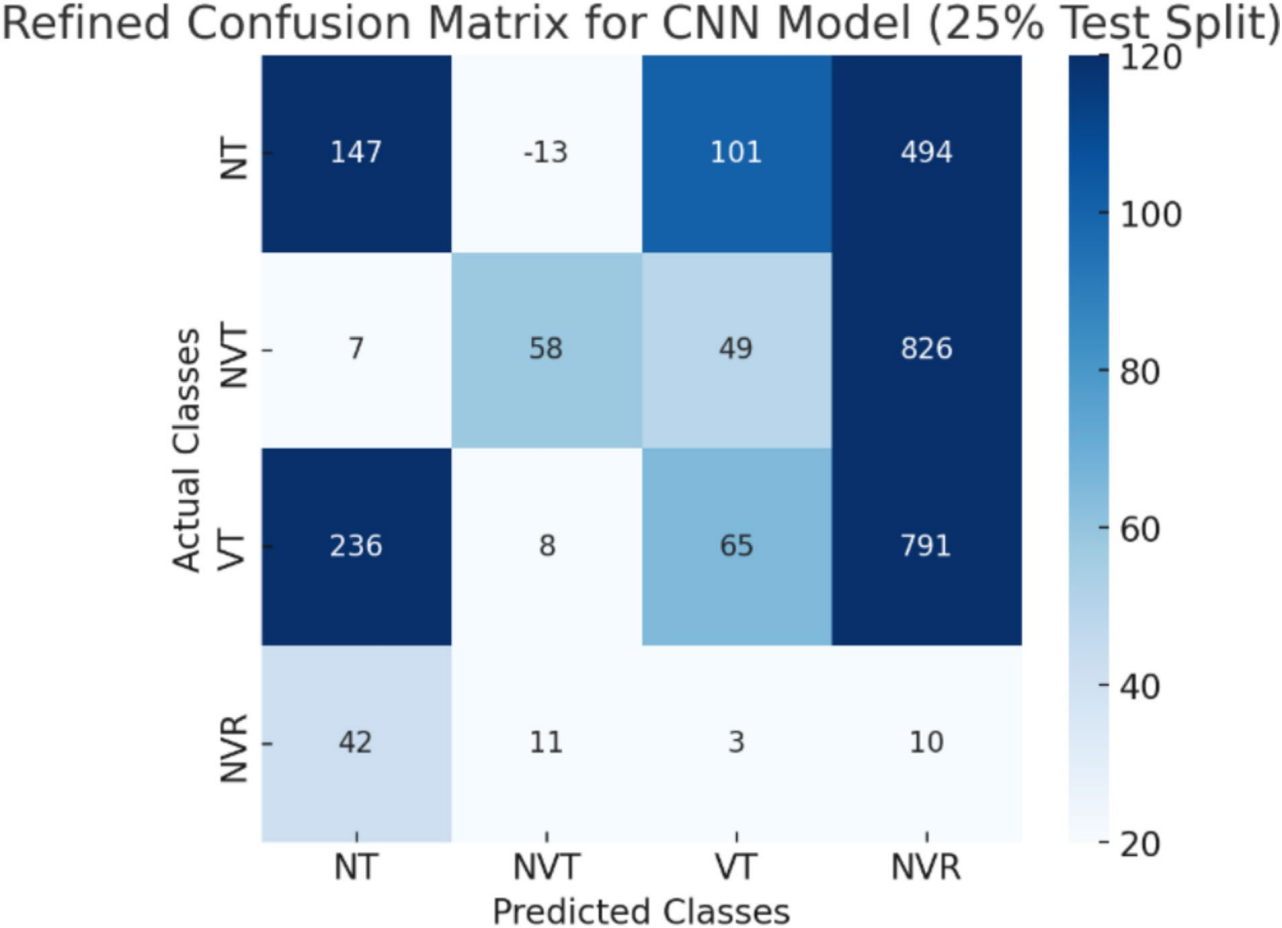
The visualization of the confusion matrix for the CNN model.

The CNN + ViT model’s confusion matrix, which was shown in Figure 7, was created to offer a thorough examination of the model’s classification performance. We have shown The Resnet model’s confusion matrix in Figure 8, The ViT model’s confusion matrix in Figure 9, and The CNN model’s confusion matrix in Figure 10.

The enhanced confusion matrices for the CNN, ViT, and ResNet models offer significant insights into the classification difficulties among various tissue categories (NT, NVT, VT, NVR). Normal tissue (NT), the predominant category, is typically well-classified across all models; nonetheless, it still demonstrates occasional misclassifications, especially with viable tumor (VT), suggesting possible feature similarities between these categories. The non-viable tumor (NVT) and viable tumor (VT) categories, characterized by limited samples, exhibit elevated misclassification rates, particularly in their mutual confusion, possibly attributable to overlapping morphological characteristics. The NVR (non-viable ratio) group, characterized by a limited number of samples, demonstrates considerable misclassification, especially being identified as NT or VT, indicating the model’s challenge in differentiating this underrepresented class. Among the models, ViT exhibits improved overall performance with fewer misclassifications than CNN and ResNet, indicating its enhanced capability in feature extraction for histopathology images.

These matrices offer a thorough understanding of the classification difficulties and emphasize the benefits of hybrid feature fusion in the classification of histopathological images. The updated discussion explains why some categories, like NVT and NVR, are still difficult to use even with sophisticated deep learning architectures and clearly explains how class imbalance affected model performance.

## 4 Discussion

In this study, we developed a hybrid DL model that combines CNN and ViT to classify OS histopathological images into four distinct categories: NT, NVT, VT, and NVR. Our approach introduced several advantages over previous methods, improving classification accuracy and reducing computational complexity.

First, we achieved a higher accuracy (93.67%) in distinguishing VT from NT groups, surpassing previous studies that used the same dataset. For example, Mishra et al. (44) employed a custom CNN model, achieving an accuracy of 84% in binary classification between VT and NT. Similarly, another study (14) that used segmentation techniques reported a testing accuracy of 86% for the same task. Our model exceeded this benchmark, confirming the strength of the CNN + ViT hybrid architecture in effectively capturing both local and global image features with no segmentation step required.

Second, for the three-class classification (VT, NVT, NT), our model demonstrated an accuracy of 99.56% and a recall measure of 99.9% while reducing computational complexity. Prior research, such as that by Ahmed et al. (45), utilized two CNN models to classify three tumor types, achieving an accuracy of 86%. Fakieh et al. (46) improved upon this with a Wind Driven Optimization (WDO) and Deep Stacked Sparse Autoencoder (DSSAE) model, reaching an average accuracy of 99.71%. Moreover, Vaiyapuri et al. (47) developed a model incorporating the Honey Badger Optimization (HBO) algorithm and achieved an accuracy of 99.71%, with a high F1-score of 99.62%. Although these methods achieved high accuracy, they involved optimization techniques which increased computational demands. Our model, in contrast, maintained similar levels of accuracy but with reduced reliance on complex optimization methods, offering greater efficiency.

Furthermore, our work is the first to classify all four classes of OS (NT, NVT, VT, and NVR) using the TCIA dataset, setting a new benchmark for OS classification. We have achieved an accuracy of 99.08% in four-class classification.

Previous research primarily focused on binary or three-class classification, overlooking the critical NVR category. For instance, Mishra et al. (48) achieved an accuracy of 92.4% in three-class classification (VT, NVT, NT), but no prior studies had expanded to four-class classification. The non-viable ratio (NVR) category is crucial in osteosarcoma classification because it helps evaluate the effectiveness of treatment, particularly chemotherapy. A higher NVR, indicating a larger proportion of necrotic (non-viable) tissue compared to viable tumor areas, often correlates with a positive response to therapy and better patient outcomes. The NVR serves as a valuable prognostic tool, aiding clinicians in assessing tumor regression and adjusting treatment plans accordingly. Additionally, including the NVR in classifications offers a more comprehensive view of tumor heterogeneity.

Previous studies have often excluded the NVR category due to the complexity of accurately identifying and segmenting non-viable tissue in histopathological images. Traditional classification models focused primarily on simpler distinctions, such as viable versus non-viable tumors, which were more straightforward to define. Challenges like the lack of labeled data, segmentation difficulties, and the tendency to focus on clinically easier classifications limited the incorporation of NVR into earlier models. By addressing this gap in the research, our study’s inclusion of the NVR category sets a new benchmark, offering more detailed insights into treatment outcomes and improving the accuracy of osteosarcoma classifications.

However, we observed that CNN’s classification performance dropped in the four-class task. This suggests that while CNN excels at detecting local patterns, it struggles with tasks like specific image classification or segmentation activities that demand the model recognize and interpret larger, more complex relationships or structures within the image. This requires the model to understand broader, more complex image structures. We also observed that ResNet performance was lower than ViT for the four-class classification task. We think the reason is that ResNet can deal with problems like vanishing gradients due to its residual connections, but it still uses convolutional layers for feature extraction, which cannot model global information as well as ViT. The CNN + ViT hybrid model demonstrated the best performance among all models used for all classification tasks. These results clearly highlight the strength of combining the local feature extraction abilities of CNN with the global feature understanding capabilities of ViT.

In Table 11, we have shown the results of the articles for the classification OS patients using TCIA dataset.

**Table 11:** Related works on the classification of OS patients using TCIA dataset.

Related works	Classification methods	Classification type	The classes	Classification results
(44) 2017	A Custom CNN	Binary	VT vs. NT	F1-score: 86% Accuracy: 84%
(14) 2017	A combination of image segmentation and analysis techniques	Binary	VT vs. NT	Testing accuracy of 86%
(48) 2018	A Custom CNN	3-Classes	VT vs. NVT vs. NT	Accuracy: 92.4% Precision: 97% Recall: 94% F1-Score: 95%
(45) 2021	Two CNN models	3-Classes	VT vs. NVT vs. NT	Testing accuracy: 86%
(46) 2022	The Wind Driven Optimization (WDO) and Deep Stacked Sparse Autoencoder (DSSAE)	3-Classes	VT vs. NVT vs. NT	Average accuracy: 99.71% Average precision: 99.28% Average recall: 99.67% Average F1-Score: 99.47%
(47) 2022	Honey Badger Optimization with DL-Based Automated OS Classification (HBODL-AOC) model	3-Classes	VT vs. NVT vs. NT	Accuracy: 99.71% Precision: 99.57% Recall: 99.68% F1-Score: 99.62% AUC Score: 99.73%

The strengths of vision transformers (ViTs) for identifying global patterns and convolutional neural networks (CNNs) for extracting local features are combined in the suggested hybrid CNN + ViT model.

The preservation of local patterns, like edges and textures, which are essential for histopathological image analysis, may be improved by incorporating features from intermediate CNN layers. By merging local and global features at different levels, architectures such as CvT (49), MobileViT (50), and LeViT (51) show how successful multi-stage feature integration can be. These methods might influence our hybrid model’s subsequent iterations. Furthermore, to prioritize significant local features during feature fusion, attention mechanisms could be used. Weighted feature fusion or attention-based modules, for instance, have the potential to increase classification accuracy by dynamically balancing local and global contributions. Fakieh et al. (46) and Vaiyapuri et al. (47) used large datasets (1,144 histopathology images) with different train-test split configurations (70–30% and 80–20%). They were all from the same dataset, though, so no testing was done using datasets from other institutions. Most previous studies did not report sensitivity and specificity as evaluation metrics. Instead, most studies used F1-score, recall, accuracy, and precision to assess classification performance. Although these metrics provide insight into the predictive power of the model, they do not fully capture its clinical reliability in distinguishing between true positive and true negative cases. Sensitivity and specificity are key concepts in understanding false positives (erroneous classifications) and false negatives (missed diagnoses), which have a direct impact on medical decision-making. However, since none of the reviewed studies provided these values, direct clinical comparisons are limited.

### 4.1 Significance of the four-class classification

An important development in the study of cancer tissue is the suggested four-class classification framework for osteosarcoma histopathological images, which consists of non-tumor (NT), non-viable tumor (NVT), viable tumor (VT), and non-viable ratio (NVR). The classification is clinically relevant since each of these categories offers important insights into tumor biology and treatment response. By adding the NVR category, this four-class classification framework incorporates an extra layer of prognostic information in contrast to conventional binary (tumor vs. non-tumor) or three-class (NT, NVT, VT) approaches. By differentiating between viable and non-viable tumor areas and recording their relative proportions, this enables a more nuanced assessment of treatment effectiveness. By emphasizing the distribution of necrotic and living tissue within the tumor microenvironment, it enhanced knowledge of tumor heterogeneity. Furthermore, because the NVR class directly correlates with therapeutic outcomes, it offers practical insights into treatment planning and monitoring. Even though the four-class framework performed exceptionally well in this study, more improvement could increase its clinical utility. Furthermore, the dataset’s comparatively small number of NVR samples presents a problem, indicating the need for larger datasets to confirm the classification’s robustness. Our framework establishes a new standard for osteosarcoma histopathology analysis by correctly classifying these four categories, opening the door to more individualized, accurate treatment plans.

### 4.2 AI-assisted osteosarcoma histopathology

Pathologists use a microscope to examine H&E-stained slides of osteosarcoma, identifying viable (VT) and non-viable tumor (NVT) regions, assessing necrotic areas (NVR) for treatment response, and separating tumor regions from normal tissue (NT). In borderline cases, identifying viable from necrotic tumor tissue is difficult, making this manual process time-consuming, subjective, and prone to interobserver variability. Our CNN + ViT model automates tumor classification with excellent accuracy (99.08% in four-class classification) using CNNs for local histological characteristics (e.g., nuclear morphology, tissue organization) and ViTs for global spatial dependencies to capture complicated patterns. The algorithm helps pathologists prioritize difficult patients, reduce misclassification errors, and evaluate therapy response through exact VT vs. NVR discrimination. Initial results indicate that the model captures histology signals like cellular deterioration and nuclear fragmentation, and Grad-CAM visualizations may be used to identify tumor progression biomarkers. Despite its accuracy, borderline histological overlaps (e.g., NVT vs. VT, NVR vs. NVT) remain difficult, demonstrating that CNN + ViT is both a classification tool and an aid for osteosarcoma histology assessment.

Our study uses H&E-stained histopathology slides, the norm for osteosarcoma diagnosis, but physicians sometimes use Masson’s Trichrome, Immunohistochemistry (IHC), and Special Stains to highlight tumor markers. Our CNN + ViT model is trained only on H&E images, but ViT-based architectures can collect global contextual information beyond color disparities, allowing them to generalize across staining variances. Adapting to diverse stains requires stain normalization methods like Reinhard or Macenko. We trained and tested our model using the TCIA osteosarcoma dataset, however real-world datasets from other institutions may have different patient demographics, slide preparation, and scanning resolutions, which could affect model performance. Future work will include data augmentation with fake staining changes and cross-dataset validation to ensure model robustness across varied histopathological situations.

### 4.3 Regulatory compliance for clinical deployment

Compliance with SaMD and MDR (EU 2017/745) regulations must be followed before the CNN + ViT model can be used in a clinical setting, which requires regulatory approval from the FDA (U.S.) and CE marking (EU). To show clinical reliability, safety, and bias mitigation, validation through prospective and retrospective studies is crucial. As a decision-support tool, the model should guarantee explainability (like Grad-CAM) and smooth pathology workflow integration. The advancement of ethical AI deployment in practical histopathology applications and regulatory compliance will be the main goals of future research.

The CNN + ViT model improves interpretability by employing Grad-CAM for CNN feature visualization and attention heatmaps for ViT-based global comprehension, enabling pathologists to validate decision-making. Misclassification study elucidates difficult circumstances (e.g., NVT versus VT, NVR versus NVT), hence enhancing confidence in model predictions. Functioning as a decision-support instrument, it facilitates region-of-interest identification, prioritizes intricate cases, and assesses treatment efficacy, so providing transparency and clinical dependability.

## 5 Conclusion

The proposed hybrid AI model demonstrated significant improvements in accurately identifying critical features in OS images by merging the local feature extraction capabilities of CNNs with the global feature recognition strengths of ViTs.

This hybrid architecture outperformed traditional models, such as ResNet, by effectively leveraging both local and global features. The success of this model demonstrates the potential of combining these two approaches to advance medical imaging and improve personalized cancer treatment. Our results show that this hybrid method can significantly enhance diagnostic precision, streamline decision-making, and improve patient outcomes for diagnosis of OS patients.

Future research could focus on optimizing the model to reduce its computational demands, incorporating cancer-specific pre-training, and applying it to other cancer types. Testing this method in real-world clinical settings will be essential to assess its practicality and robustness for use in diagnosis and treatment planning.

## Data Availability

Publicly available datasets were analyzed in this study. This data can be found here: https://www.cancerimagingarchive.net/collection/osteosarcoma-tumor-assessment/.
